# Perlecan deficiency causes endothelial dysfunction by reducing the expression of endothelial nitric oxide synthase

**DOI:** 10.14814/phy2.12272

**Published:** 2015-01-27

**Authors:** Risa Nonaka, Takafumi Iesaki, Susana de Vega, Hiroyuki Daida, Takao Okada, Takako Sasaki, Eri Arikawa‐Hirasawa

**Affiliations:** Research Institute for Disease of Old Age, Juntendo University Graduate School of Medicine, Tokyo, Japan; Department of Physiology, Juntendo University Graduate School of Medicine, Tokyo, Japan; Department of Cardiovascular Medicine, Juntendo University Graduate School of Medicine, Tokyo, Japan; Department of Biochemistry, Faculty of Medicine, Oita University, Oita, Japan

**Keywords:** Endothelial cell, nitric oxide synthase, perlecan

## Abstract

Perlecan is a major heparan sulfate proteoglycan found in the subendothelial extracellular matrix of the vascular wall. The aim of this study was to investigate the role of perlecan in the regulation of vascular tone. A previously developed conditional perlecan‐deficient mouse model was used to measure changes in the isometric force of isolated aortic rings. The vessels were first precontracted with phenylephrine, and then treated with increasing concentrations of vasorelaxants. Endothelium‐dependent relaxation, elicited by acetylcholine, was significantly reduced in the perlecan‐deficient aortas, whereas endothelium‐independent relaxation caused by the exogenous nitric oxide donor sodium nitroprusside remained well preserved. The expression of the endothelial nitric oxide synthase (eNOS) gene, detected by real‐time polymerase chain reaction, was significantly decreased in the perlecan‐deficient aortas. The expression of eNOS protein detected using Western blotting was also significantly decreased in the perlecan‐deficient aortas. We examined the role of perlecan in eNOS gene expression by creating perlecan knockdown human aortic endothelial cells using small interfering RNA (siRNA) for perlecan. Perlecan gene expression was significantly reduced in the perlecan siRNA‐treated cells, resulting in a significant decrease in eNOS gene expression. Perlecan deficiency induced endothelial dysfunction, as indicated by a reduction in endothelium‐dependent relaxation due, at least partly, to a reduction in eNOS expression. These findings suggest that perlecan plays a role in the activation of eNOS gene expression during normal growth processes.

## Abstract (precis)

Perlecan deficiency induced endothelial dysfunction at least partly, to a reduction in eNOS expression. These findings suggest that perlecan plays a role in the activation of the eNOS expression during normal growth processes.

## Introduction

Vascular endothelial cells and vascular smooth muscle cells are two major research targets in the field of vascular physiology. The extracellular matrix in the vascular wall has recently attracted attention, as it not only provides structural support, but also modulates several cellular functions, including cellular adhesion, proliferation, differentiation, and development (Pillarisetti 2000; Jiang and Couchman 2003; Iozzo 2005). The basement membrane is a thin sheet of the extracellular matrix that underlies the vascular endothelium and surrounds smooth muscle cells. Some of the key constituents of the subendothelial extracellular matrix are heparan sulfate proteoglycans (HSPGs). Perlecan (HSPG2) is a major HSPG in the basement membrane, with a molecular weight of over 400 kDa and a protein core consisting of five domains. It interacts with a number of extracellular matrix molecules, including laminin, collagen IV, fibronectin, fibrillin, and several growth factors, such as vascular endothelial growth factor (VEGF), fibroblast growth factors (FGFs), and platelet‐derived growth factor (PDGF) (Iozzo 2005). Perlecan also interacts with plasma lipoprotein very low density lipoprotein (VLDL) (Hummel et al. 2004) and integrin cell surface receptor (Hummel et al. 2004). It also plays a role in a number of processes in the vasculature, including atherosclerosis, tumor angiogenesis, smooth muscle cell activity modulation, endothelial proliferation, and vascular development (Segev et al. 2004).

Others and we have previously reported that the homozygous deletion of *HSPG2* (*HSPG2*^*−/−*^) in mice results in lethal chondrodysplasia and in cardiovascular abnormalities, such as transposition of the great arteries (Arikawa‐Hirasawa et al. 1999; Costell et al. 1999, 2002). Perlecan deficiency (*HSPG2*^*−/−*^) also causes perinatal lethal chondrodysplasia in humans (Costell et al. 1999; Arikawa‐Hirasawa et al. 2001), suggesting a crucial role for perlecan in the development of cartilage and in the cardiovascular system. Mutations in the perlecan gene (*HSPG2)* have been identified in patients with Schwartz–Jampel syndrome, a nonlethal condition characterized by myotonia and mild chondrodysplasia (Nicole et al. 2000; Arikawa‐Hirasawa et al. 2002; Stum et al. 2006). We investigated the role of perlecan in adult organs using a lethality rescued perlecan‐null mouse model expressing recombinant perlecan specifically in cartilage (*HSPG2*^*−/−*^*‐Tg*) (Xu et al. 2010; Inomata et al. 2012; Ishijima et al. 2012; Kaneko et al. 2013). In addition to our lethality rescued *HSPG2*^*−/−*^*‐Tg* mouse model, studies employing mutant mice, such as heterozygous perlecan knockout (*HSPG2*^*+/−*^) mice (Vikramadithyan et al. 2004) or heparan sulfate (HS)‐deficient perlecan expressing mice (Tran‐Lundmark et al. 2008), have provided further insights into the in vivo function of perlecan in adult tissues.

Endothelial dysfunction is considered to be a key variable in the pathogenesis of atherosclerosis and in its complications (Bonetti et al. 2002). The dysfunctional condition includes reduction in nitric oxide (NO) bioavailability, which may result in reduced vasorelaxation, thrombus formation, deposition of serum lipids, and the migration and proliferation of vascular smooth muscle cells (VSMCs), which leads to the formation of stenotic lesions in blood vessels (Cai and Harrison 2000). The regulation of vascular tone is the most widely studied aspect of the endothelial function, and NO is the major contributor to endothelium‐dependent relaxation in large arteries. In the present study, we investigated the effects of perlecan deletion on endothelium‐dependent vascular relaxation during the normal growth process and explored the mechanisms underlying the effects.

## Materials and Methods

### Animals

Perlecan‐null (*HSPG2*^*−/−*^*)* mice die perinatally due to premature cartilage development (Arikawa‐Hirasawa et al. 1999; Costell et al. 1999). We previously created a perlecan transgenic mouse line (WT‐Tg, *HSPG2*^+/+^; *COL2A1*‐*HSPG2*^*Tg/−*^*),* which expresses recombinant perlecan in cartilage, using a cartilage‐specific *COL2A1* promoter/enhancer (Tsumaki et al. 1999) to restore cartilage abnormalities. Subsequently, we created lethality‐rescued mice (*HSPG2*^*−/−*^ ‐Tg, *HSPG2*^*−/−*^;*COL2A1*‐*HSPG2*^*Tg/−*^*),* by mating the transgenic mice with heterozygous *HSPG2*^*+/−*^ mice (Xu et al. 2010). We maintained these mice on a mixed genetic background of C57BL/6 and 129SvJ. In the present study, we used *HSPG2*^*−/−*^ ‐Tg mice for the experimental group and WT‐Tg (*HSPG2*^+/+^; *COL2A1*‐*HSPG2*^*Tg/−*^*),* mice (10 weeks of age) for the control group. All experimental procedures were performed in accordance with the guidelines for the care and use of animals at Juntendo University, Tokyo, Japan.

### Measurement of changes in force in the mice aorta

The mice were sacrificed under anesthesia (Pentobarbital; 50 mg/kg, intraperitoneal administration). The descending thoracic aorta was isolated and cut into transverse rings (~2 mm in length), which were used to measure the changes in force. Care was taken not to touch the endothelial surface in order to preserve the functional endothelium. The techniques used to measure the changes in force were adapted from previously described methods (Iesaki et al. 1999; Sumiyoshi et al. 2008). Briefly, aortic rings were mounted on wire hooks attached to force displacement transducers (Nihon Kohden, Tokyo, Japan) and changes in the isometric force were recorded on a thermal recorder (Rika Denki, Tokyo, Japan). The rings were incubated in individually thermostated (37°C) 10‐mL baths filled with oxygenated Krebs bicarbonate buffer (118 mmol/L NaCl, 4.7 mmol/L KCl, 1.5 mmol/L CaCl_2_, 25 mmol/L NaHCO_3_, 1.1 mmol/L MgSO_4_, 1.2 mmol/L KH_2_PO_4_, and 5.6 mmol/L glucose at pH 7.4). An optimal passive tension of 0.5 g was applied to the rings throughout the experiment. The vascular rings were initially exposed to high‐K^+^ Krebs bicarbonate buffer, containing 60 mmol/L KCl in place of NaCl to produce maximal force and to enhance the reproducibility of subsequent contractions. After a wash out of high‐K^+^ buffer, the vessels were submaximally contracted with 1 *μ*mol/L phenylephrine. Once a steady‐state level of contraction was achieved, endothelial‐dependent relaxation and endothelial‐independent relaxation were elicited by the administration of increasing concentrations of acetylcholine (ACh) and sodium nitroprusside (SNP), respectively. Relaxation was expressed as the percent change in the steady‐state level of contraction. Comparisons between groups were made with two‐way reported measure ANOVA.

### Quantitative real‐time polymerase chain reaction

Total RNA was isolated from mouse thoracic aortic tissue or human aortic endothelial cells (HAECs; LONZA, Walkersville, MD) using TRIzol reagent (Life Technologies, Carlsbad, CA), according to the manufacturer's instructions. cDNA was generated from 1 *μ*g of total RNA with M‐MLV reverse transcriptase (Promega, Madison, WI) and a random primer (TAKARA, Siga, Japan). SYBR Green was used for detection, and RNA expression was normalized to that of the housekeeping gene *β*‐actin for mouse, GAPDH for human. In the graph, the eNOS, von Willebrand factor, and perlecan (HSPG2) expression levels are indicated as relative to *β*‐actin or GAPDH. The PCR reaction was carried out in an ABI Prism^®^ 7500 Fast Sequence Detection System (Life Technologies). The primer sequences were as follows: For the mouse tissue, mouse eNOS forward 5′‐CTGGCAGCCCAAGACCTA‐3′, mouse eNOS reverse 5′‐GTGACATCGCCGCAGACAA‐3′, mouse von Willebrand factor forward 5′‐GATGCCCCAGTCAGCTCTAC‐3′, mouse von Willebrand factor reverse 5′‐ TCAGCCTCGGACAACATAGA‐3′, mouse *β*‐actin forward 5′‐TGGAATCCTGTGGCATCCATGAAAC‐3′, mouse *β*‐actin reverse 5′‐TAAAACGCAGCTCAGTAACAGTCCG‐3′. For the HAECs, human HSPG2 forward 5′‐GGCTGAGGGCATACGATGGCT‐3′, human HSPG2 reverse 5′‐CCCACTGCCCAGGTCGTCTCC‐3′, human eNOS forward 5′‐CCCTTCAGTGGCTGGTACAT‐3′, human eNOS reverse 5′‐CACGATGGTGACTTTGGCTA‐3′, human GAPDH forward 5′‐ACCACAGTCCATGCCATCAC‐3′, human GAPDH reverse 5′‐TCCACCACCCTGTTGCTGTA‐3′

### Western blotting

Thoracic aortic tissue was isolated and then homogenized in cold lysis buffer, and the lysate was centrifuged at 16,000 *g* for 15 min at 4°C. The lysis buffer contained 50 mmol/L Tris‐HCl (pH 7.2), 150 mmol/L NaCl, 1% Nonidet P‐40, 1% sodium deoxycholate, 0.1% SDS containing protease, and phosphatase inhibitor cocktails (Complete Protease Inhibitor Cocktail and PhosSTOP; Roche, Rotkreuz, Switzerland). The protein concentration was determined using a BCA protein assay kit (Thermo Scientific, Rockford, IL) and then solubilized in NuPAGE^®^ LDS sample buffer (Life Technologies) containing dithiothreitol. The samples (15 *μ*g/lane) were resolved via electrophoresis on 4–12% SDS‐PAGE gels, and then transferred to a PVDF membrane (Life Technologies, Carlsbad, CA). After blocking with PVDF blocking reagent (TOYOBO, Osaka, Japan), the membrane was incubated with primary antibodies in blocking reagent overnight. After washing, the membrane was incubated with horseradish peroxidase (HRP)‐conjugated secondary antibodies in blocking reagent and visualized with SuperSignal^®^West Dura Extended Duration Substrate (Thermo Scientific, Rockford, IL). Specific bands were quantitated using the ImageJ software program. In the graph, the eNOS protein level is indicated as relative to *β*‐actin. The experiments were performed at least three times using different sibling pairs of animals. The antibodies were prepared as follows: the primary antibodies, mouse anti‐eNOS/NOS type III antibodies (BD Biosciences, Franklin Lakes, NJ) or mouse anti‐*β*‐actin antibody (Santa Cruz Biotechnology, Inc., Dallas, TX) were diluted at 1:1000 or 1:5000 in blocking reagent. The secondary antibodies, anti‐mouse IgG HRP‐conjugated secondary antibodies (GE Healthcare, Little Chalfont, UK) were diluted at 1:5000 in blocking solution.

### Cell culture and small interfering RNA (siRNA)

Human aortic endothelial cells (HAECs) were grown using the Endothelial Cell Growth Media Kit (EGM‐2 BulletKit; LONZA), and plated at a density of 2 × 10^5^ cells on 6‐well plates. Perlecan siRNA and control siRNA were purchased from Santa Cruz Biotechnology. HAECs were transfected with either perlecan or control siRNA, according to the manufacturer's recommendations. Forty‐eight hours after transfection, knockdown efficiency was assessed using quantitative RT‐PCR (qPCR), as described above. For perlecan rescue experiments, we used the recombinant perlecan protein (rPerlecan) kindly provided by Dr. Sasaki. The recombinant perlecan was purified from the condition media of 293 cells transfected with a perlecan cDNA expression vector (Noonan et al. 1991; Costell et al. 1997; Xu et al. 2010). The full‐length perlecan cDNA (Ishijima et al. 2012) was cloned into the PCEP4‐Mul‐PURD expression vector (Hozumi et al. 2006), which contains sequences for the CMV promoter, multiple cloning sites, BM40 signal peptide (Hozumi et al. 2006). A twenty‐four well tissue culture plate was coated with rPerlecan (20 *μ*g/mL) in PBS, including 1 mmol/L CaCl_2_ and 0.5 mmol/L MgCl_2_ at 4°C for 48 h. After the plate was washed twice with PBS, HAECs treated with Perlecan siRNA or Control siRNA, as described above, were plated at density of 3 × 10^5^ cells on the Perlecan or a control plate and cultured for 36 h. The eNOS RNA expression was analyzed using qPCR.

### Heparinase III digestion of HAECs

Cell surface heparan sulfate chains of HAECs were digested by heparinase III as described method (Kerever et al. 2007) with some modifications. HAECs were incubated with 5 mU/mL of heparinase III (Sigma‐Aldrich, St. Louis, MO) in 50 mmol/L HEPES buffer (pH 7.0), containing 100 mmol/L NaCl and 1 mmol/L CaCl_2_ at 37°C for 1 h. After the incubation, the heparinase III solution was removed and the cells were cultured with the growth media. We confirmed successful heparinase III digestion using immunostaining. A time course of the eNOS RNA expression was performed using qPCR as described above.

### Immunostaining

HAECs were plated at density of 3 × 10^5^ cells on Type‐1 collagen‐coated 8 well chambers and cultured for 48 h. After heparinase III digestion for 1 h, the cells were fixed immediately (time point 0) or 24 h later (time point 24 h) with 4% paraformaldehyde at room temperature. Nonspecific binding was blocked with 0.2% gelatin/PBS for 10 min and cells were incubated with anti‐heparan sulfate antibody (10E4 epitope) antibody or anti‐Δheparan sulfate (3G10 epitope) antibody (Seikagaku Corporation, Tokyo, Japan) at 1:400, and anti‐perlecan (clone A7L6) (Chemicon, Temecula, CA) antibody at 1:400 in 0.2% gelatin/PBS at 4°C overnight. Following a wash with PBS, the cells were incubated with goat anti‐mouse IgM alexafluor 488 and goat anti‐rat IgG alexafluor 546 (Molecular Probes, Invitrogen Corporation, Carlsbad, CA) at 1:400 in 0.2% gelatin/PBS for 1 h. The cells were washed and then incubated for 10 min in bis‐benzimide (1:5000, Molecular Probes, Invitrogen Corporation). After extensive washes, cells were mounted in fluoro‐gel with Tris buffer (Electron Microscopy Sciences, Hatfield, PA). Images were taken using a Leica TCS‐SP5 LSM confocal microscope.

### Statistical analysis

The data are presented as the mean ± SEM. Comparisons between groups were made with two‐way reported measure ANOVA and with the unpaired *t*‐test. A *P* value of <0.05 was considered to be statistically significant.

## Results

### *HSPG2* deletion decreases the relaxation of mouse aorta

**Figure 1.**
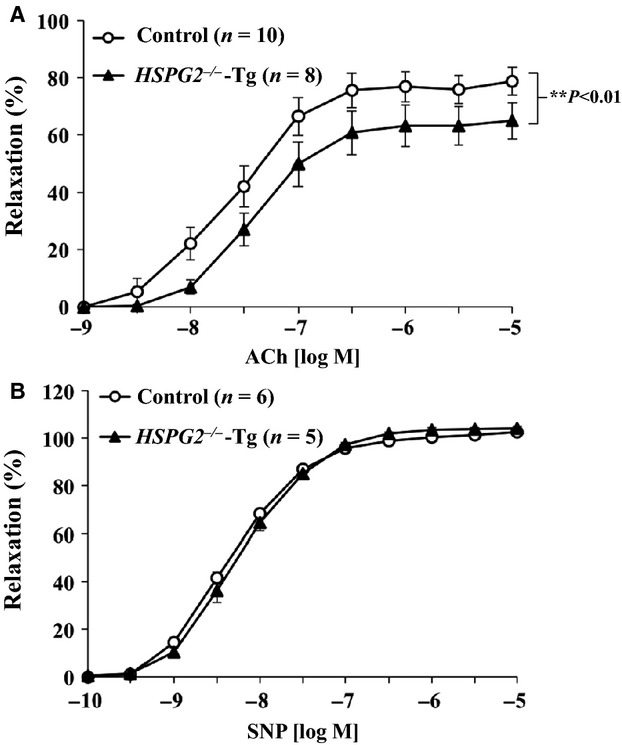
*HSPG2*^*−/−*^‐Tg aortas have a reduced relaxation in response to ACh or SNP. The aortic rings were precontracted with 1 *µ*mol/L phenylephrine (PE), and increasing concentrations of (A) acetylcholine (ACh) or (B) sodium nitroprusside (SNP) were added. The degree of relaxation is expressed as the percent relaxation of the PE‐induced tone (The bars indicate the mean ± SEM,* n* = 5–10).

An endothelium‐dependent relaxation of the mouse aorta was elicited by ACh. Aortic relaxation in response to ACh was significantly reduced in the *HSPG2*^*−/−*^‐Tg aortas compared to that of the control aortas, resulting in the downward shift of the concentration‐response curve (Fig. 1A). On the other hand, the endothelium‐independent relaxation elicited by exogenous nitric oxide donor SNP was not affected by the deletion of *HSPG2*, as shown in Figure 1B, indicating that the sensitivity of vascular smooth muscle to exogenous nitric oxide donors is well preserved, even in *HSPG2*^*−/−*^‐Tg aortas.

### HSPG2 deletion decreases the expression level of eNOS in mouse aortas

**Figure 2.**
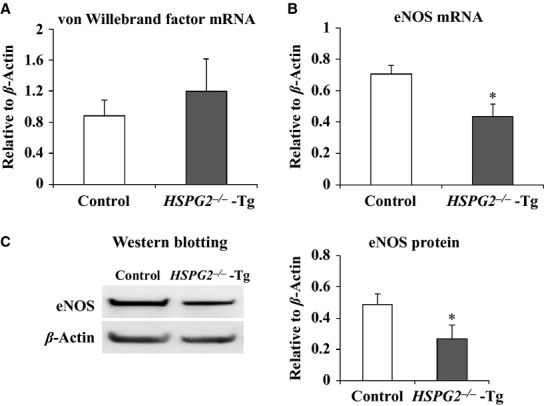
Expression of endothelial nitric oxide synthase (eNOS) in the *HSPG2*^*−/−*^‐Tg aorta. RNA expression levels of von Willebrand factor and eNOS in the aortic tissues of 10 week control and *HSPG2*^*−/−*^‐Tg mice were analyzed using qPCR. (A) The expression of von Willebrand factor was not significantly different, while (B) eNOS expression was significantly reduced in the *HSPG2*^*−/−*^‐Tg animals (*n* = 6 per genotype. The bars indicate the mean ± SEM). RNA expressions levels were normalized to that of *β*‐actin and were indicated as relative to *β*‐actin. (C) The protein expression levels of eNOS and *β*‐actin in the aortic tissues were evaluated, using Western blotting. Each band was quantified using ImageJ software and is shown as relative to *β*‐actin. The protein expression levels of eNOS were significantly decreased in the HSPG2^−/−^‐Tg mice compared to that in the control mice (*n* = 4 per genotype. The bars indicate the mean ± SEM). *P < 0.05 versus control mice.

We explored the mechanisms underlying the reduction of endothelium‐dependent relaxation by measuring RNA expression levels of both eNOS and von Willebrand factor (vWF), an endothelial cell specific gene, using qPCR. RNA was extracted from aortic tissue from control and from *HSPG2*^*−/−*^‐Tg animals. The vWF expression level in the control and in the *HSPG2*^*−/−*^‐Tg aortas was not significantly different (Fig. 2A). However, qPCR analysis revealed that eNOS mRNA expression was significantly reduced in the *HSPG2*^*−/−*^‐Tg aortas (Fig. 2B). We also measured protein expression levels of eNOS in the control and in the *HSPG2*^*−/−*^‐Tg aortas by Western blotting. The protein level of eNOS was significantly decreased in the *HSPG2*^*−/−*^‐Tg aortas compared with that of the control aortas (Fig. 2C). These results indicated that eNOS expression was decreased in the perlecan‐deficient aortas.

### eNOS expression was decreased in HAECs by Perlecan siRNA treatment

**Figure 3.**
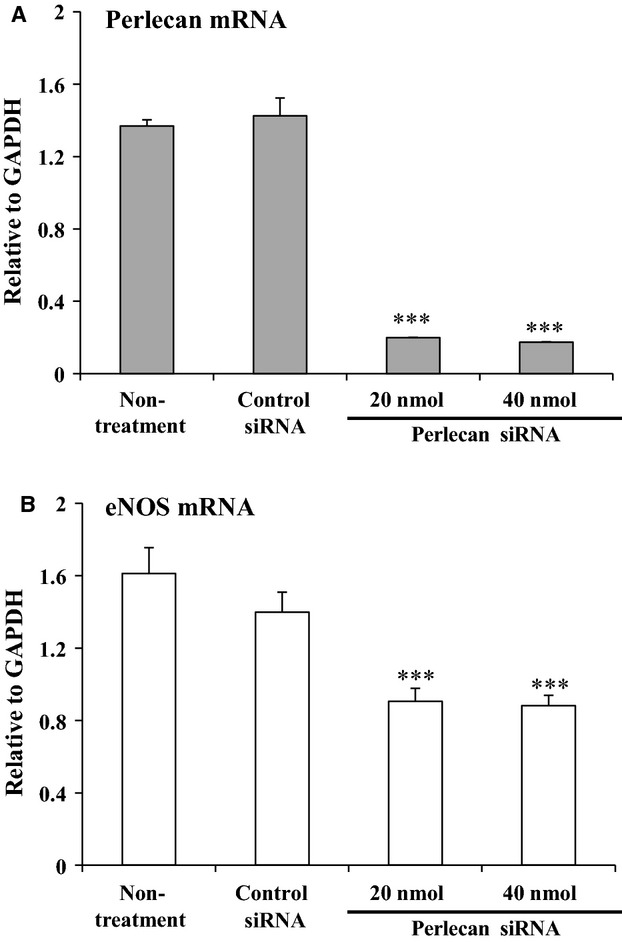
RNA expression of eNOS in human aortic endothelial cells (HAECs) treated with *HSPG2* siRNA. Analyses of perlecan and eNOS RNA expression levels in the HAECs treated with or without control siRNA and 20 or 40 nmol of Perlecan siRNA using qPCR. RNA expressions were normalized to that of GAPDH and are indicated as relative to GAPDH. (A) Perlecan expression in HAECs was significantly decreased by approximately 90% following Perlecan siRNA treatment. (B) eNOS expression showed a significant decrease of approximately 50% following treatment with Perlecan siRNA. The bars indicate the mean ± SEM (*n* = 3), ***P < 0.001 versus control cells.

We examined the relationship between eNOS expression and perlecan expression by creating perlecan knockdown HAECs in culture using Perlecan siRNA treatment. We measured the RNA expression levels by qPCR using RNA extracted from HAECs treated with control or Perlecan siRNA. We confirmed that perlecan mRNA expression levels in HAECs were significantly decreased by approximately 90% following treatment with both 20 and 40 nmol of Perlecan siRNA (Fig. 3A). The eNOS mRNA expression was significantly decreased by approximately 50% following Perlecan siRNA treatment (Fig. 3B). These results indicated that reduced perlecan expression decreases eNOS expression in HAECs.

### Depletion of heparan sulfate chains does not affect the eNOS expression

**Figure 4.**
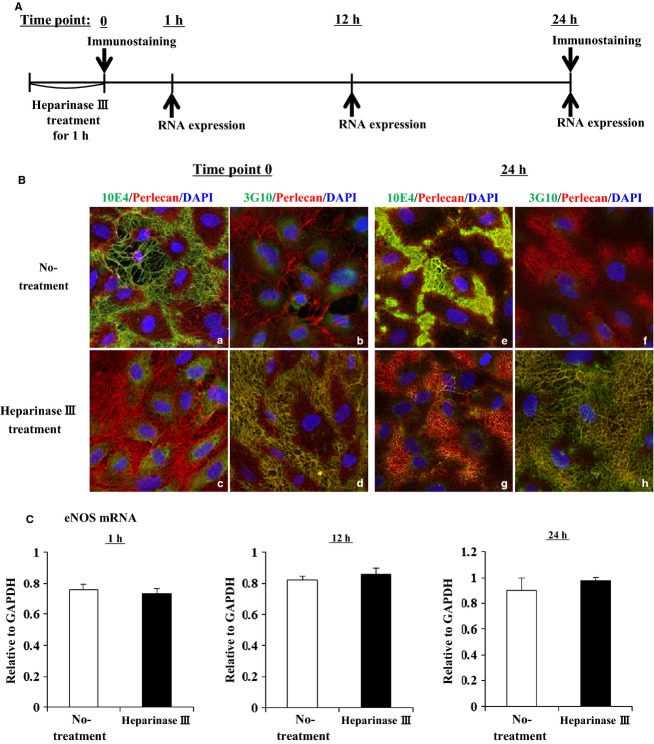
RNA expression levels of eNOS in HAECs treated with heparinase III. (A) A time course of experimental protcol using heparitinase III. (B) The heparan sulfate chains were removed from HAECs using heparinase III. After a 1 h treatment with or without heparinase III, the cells were fixed immediately (time point 0) or 24 h later (time point 24 h), and immunostaining was performed with 10E4 or 3G10 (Green), and perlecan (Red) antibodies. Successful heparinase III digestion is indicated by negative staining of 10E4 (c and g) and positive staining of 3G10 (d and h). At 24 h culture after heparinase III treatment, heparan sulfate chains were not detected (g and h). (C) Analysis of eNOS RNA expression levels in HAECs treated with or without heparinase III using qPCR. eNOS RNA expressions was normalized to that of GAPDH and it is indicated as relative to GAPDH. eNOS expression levels in the heparinase III treated HAECs was not significantly different compared with that of the non‐treated HAECs at 1, 12, and 24 h later. The bars indicate the mean ± SEM (*n* = 3)

We performed enzymatic depletion of the heparan sulfate chains of perlecan on HAECs to explore whether heparan sulfate chains are critical for eNOS expression. The time course of the experimental protocol is shown in Figure 4A. First, we confirmed the depletion of heparan sulfate chains in HAECs by immunostaining using 10E4 and 3G10 antibodies. After heparinase III treatment for 1 h (time point 0), the staining by 10E4 antbody (green), which is directed against heparan sulfate chains, was not detected (Fig. 4Ba and Bc) and the staining by 3G10 antibody (green), which is directed against heparinase‐generated HS stubs, was detected in the treated but not untreated HAECs (Fig. 4Bb and Bd). Similar results were obtained after 24 h (time point 24 h) (Fig. 4Be–Bh). In these conditions, we analyzed eNOS expression by qPCR at 1, 12, and 24 h after heparinase III treatment for 1 h. The eNOS expression levels in the heparinase treated HAECs were not different compared with that of the non‐treated HAECs at any time point (Fig. 4C). These results suggest that the decrease of eNOS expression in perlecan‐deficient aortas is not due to the heparan sulfate chains.

### Perlecan protein is necessary for eNOS expression

**Figure 5.**
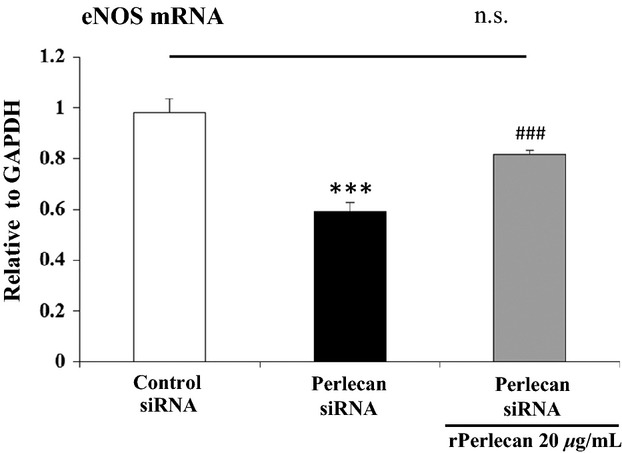
RNA expression level of eNOS in Perlecan knockdown HAECs on perlecan recombinant protein. qPCR analysis of eNOS RNA expression level in HAECs treated with Perlecan or Control siRNA and seeded on a plate coated with or without perlecan recombinant protein (rPerlecan). The level of the eNOS RNA expression was normalized to that of GAPDH and it is indicated as relative to GAPDH. The eNOS expression level in HAECs treated with Perlecan siRNA was significantly decreased compared to that of Control siRNA treated HAECs. HAECs treated with Perlecan siRNA and plated on rPerlecan was no significantly different compared with that of Control siRNA treated HAECs. The bars indicate the mean ± SEM (*n* = 4) ***P < 0.001 versus Control siRNA treated HAECs without rPerlecan, ^###^*P* < 0.001 versus Perlecan siRNA treated HAECs without rPerlecan.

In order to explore whether perlecan protein is critical for eNOS expression, we performed perlecan rescue experiments using recombinant perlecan protein (rPerlecan). HAECs treated with Perlecan siRNA or Control siRNA were seeded on plates coated with or without rPerlecan. After 36 h, the eNOS expression level was analyzed by qPCR. First, we confirmed that the eNOS expression level in HAECs treated with Perlecan siRNA was significantly decreased compared with that of Control siRNA. However, the eNOS expression level in HAECs treated with Perlecan siRNA was restored to the level almost similar to that in HAECs treated with Control siRNA by rPerlecan (Fig. 5). These results suggest that perlecan is responsible for the decrease in eNOS expression in perlecan‐deficient aortas.

## Discussion

In the present study, we showed that a deficiency of perlecan results in the impairment of endothelium‐dependent vascular relaxation in mice aorta, whereas endothelium‐independent relaxation in response to the nitric oxide donor SNP remained well preserved. We investigated the mechanism(s) underlying the reduction in endothelium‐dependent relaxation by examining the eNOS expression levels in aortic tissue and found that both eNOS mRNA and protein levels were decreased in the perlecan‐null aortas. We further examined the relationship between perlecan deficiency and a decreased eNOS expression by treating HAECs with perlecan siRNA and found that a reduction in the perlecan gene expression induced a decrease in eNOS gene expression. This is the first report to show that perlecan deficiency results in a reduction in endothelium‐dependent relaxation due, at least partly, to a decrease in eNOS expression. Although perlecan has been implicated in vascular development, the function of VSMCs with respect to relaxation is not affected by the deletion of the perlecan gene, suggesting a lesser contribution of perlecan in VSMCs than in endothelial cells.

NO is a gaseous lipophilic free radical generated by constitutively expressed eNOS in vascular endothelial cells (Braam and Verhaar 2007). The expression levels of eNOS are altered in patients with various pathophysiological conditions, such as cardiovascular disease, atherosclerosis, diabetes mellitus, and hypertension (Chatterjee et al. 2008). The transcriptional activation of the eNOS gene is stimulated by shear stress (Papapetropoulos et al. 1999), exercise (Sessa et al. 1994), and the action of lysophosphatidylcholine (Zembowicz et al. 1995) and several growth factors, including VEGF (Bouloumie et al. 1999), bFGF, and epidermal growth factor (EGF) (Braam and Verhaar 2007). Conversely, eNOS expression is down‐regulated by tumor necrosis factor‐alpha (TNF‐*α*) (Nishida et al. 1992), hypoxia (McQuillan et al. 1994), and high concentrations of low‐density lipoprotein (LDL) (Laufs et al. 1998). eNOS activity at the post‐translational level is regulated by several mechanisms, including interactions with other proteins, acylation, phosphorylation, and cellular localization (Braam and Verhaar 2007). In the present study, we demonstrated that perlecan plays a role in endothelium‐dependent vascular relaxation, acting in part through maintenance of the eNOS expression levels.

Proliferation of endothelial cells requires multiple growth factors, including VEGF and FGF‐2 (Carmeliet 2000; Iozzo and San Antonio 2001), which elicit their activities by binding to HSPGs in the vascular wall (Iozzo and San Antonio 2001). These growth factors must bind to HSPGs in the vascular wall in order to function stably (Iozzo and San Antonio 2001). These growth factors also upregulate eNOS expression (Braam and Verhaar 2007), therefore it is conceivable that perlecan deficiency results in reduced binding of growth factors to the vessel wall, thereby reducing eNOS expression. The expression of vWF, another marker of endothelial cells, was not affected by perlecan deficiency in this study, suggesting that the reduction in eNOS expression is rather specific to perlecan deficiency. Perlecan binds to several growth factors, including VEGF and FGF‐2, via its HS chains (Zoeller et al. 2009). Perlecan from endothelial cells and the recombinant endorepellin protein, a C‐terminal fragment of perlecan, binds to VEGFR‐1 and ‐2, and modulate the VEGF‐VEGFR signaling pathway in endothelial cells (Goyal et al. 2011; Ishijima et al. 2012). In this study, we showed that the depletion of heparan sulfate chains did not affect the eNOS expression level in HAECs. On the other hand, the eNOS expression level of HAECs treated with Perlecan siRNA was restored to the similar level of Control siRNA in the presence of recombinant perlecan protein. These results suggest that the decrease in the eNOS expression level in perlecan knockout aorta is due to the deficiency of the perlecan core protein. The precise mechanisms that underlie this reduced eNOS expression induced by perlecan deficiency remain to be elucidated. However, this study is the first, to our knowledge, to show a direct relationship between perlecan deficiency and a reduction in eNOS mRNA expression.

We previously reported that heterozygous deficiency of perlecan results in a reduced rate of atherosclerosis in apoE null mice (Vikramadithyan et al. 2004), suggesting that perlecan possesses pro‐atherosclerotic properties. In addition, perlecan heparan sulfate (HS) chains promote atherosclerosis, as the depletion of endogenous perlecan HS was associated with a reduced frequency of atherosclerosis in apoE null mice (Tran‐Lundmark et al. 2008), again suggesting that perlecan is pro‐atherosclerotic. In contrary, our results suggest that the perlecan protein plays an atheroprotective role by activating the expression of eNOS during the normal growth process. The discrepancy in these results may be due to the differences in the animal models used and the time points of observation in each study, as the formation of atherosclerotic lesions is a chronic process that includes multiple steps of progression over several weeks. In the present study, we examined the role of perlecan in normal growth, not in animals exposed to atherosclerotic stimuli.

We previously investigated the effects of perlecan deletion on several adult organs in mice, including skeletal muscle (Xu et al. 2010), corneal epithelial tissues (Inomata et al. 2012), endochondral bone formation (Ishijima et al. 2012), and synovial joints in the setting of knee osteoarthritis (Kaneko et al. 2013). Taken together, the results suggested that perlecan plays diverse roles in supporting tissues and homeostasis of tissue functions. In the present study, we found that the deletion of perlecan resulted in endothelial dysfunction, which is a new and rather unexpected finding, despite previous reports indicating that perlecan plays a role in the development of the cardiovascular system (Costell et al. 2002). This finding may indicate a potential cardiovascular risk in patients with Schwartz–Jampel syndrome, a disease caused by mutations in the perlecan gene in humans (Nicole et al. 2000; Arikawa‐Hirasawa et al. 2002; Stum et al. 2006).

In conclusion, we showed that deficiency of perlecan led to endothelial dysfunction, as represented by a reduction in endothelium‐dependent relaxation, which is thought to constitute the very early phase of atherosclerosis (Vanhoutte 2009). This dysfunction was due, at least partly, to a reduction in eNOS expression, indicating that perlecan plays a role in the activation of eNOS gene expression during normal growth.

## Acknowledgments

We specially thank Dr. Y.Yamada for valuable advice, and Dr. Z. Xu, Dr. N. Liang, Dr. A. Kerever, Dr. T. Nakamura, and Dr. K. Sumiyoshi for the mouse management and technical assistance.

## Conflict of Interest

None declared.
